# A T‐cell diagnostic test for cystic echinococcosis based on Antigen B peptides

**DOI:** 10.1111/pim.12499

**Published:** 2017-11-24

**Authors:** L. Petrone, V. Vanini, M. Amicosante, A. Corpolongo, M. A. Gomez Morales, A. Ludovisi, G. Ippolito, E. Pozio, A. Teggi, D. Goletti

**Affiliations:** ^1^ Translational Research Unit Department of Epidemiology and Preclinical Research “L. Spallanzani” National Institute for Infectious Diseases (INMI) Rome Italy; ^2^ Department of Biomedicine and Prevention University of Rome “Tor Vergata” Rome Italy; ^3^ ProxAgen Ltd Sofia Bulgaria; ^4^ Clinical Department National Institute for Infectious Diseases (INMI) Rome Italy; ^5^ Department of Infectious, Parasitic and Immunomediated Diseases Istituto Superiore di Sanità (ISS) Rome Italy; ^6^ Scientific Direction National Institute for Infectious Diseases (INMI) Rome Italy; ^7^ Department of Infectious and Tropical Diseases Sant'Andrea Hospital University of Rome “Sapienza” Rome Italy

**Keywords:** cytokine, *Echinococcus* spp, ELISA/enzyme‐linked immunosorbent assay, hydatidosis, immunodiagnosis

## Abstract

Cystic echinococcosis (CE) immunodiagnosis is still imperfect. We recently set‐up a whole‐blood test based on the interleukin (IL)‐4 response to the native Antigen B (AgB) of *Echinococcus granulosus*. However, AgB is encoded by a multigene family coding for five putative subunits. Therefore, the aims of this study were to analyse the IL‐4 response to peptides spanning the immunodominant regions of the five AgB subunits and to evaluate the accuracy of this assay for CE diagnosis. Peptides corresponding to each subunit were combined into five pools. A pool containing all peptides was also used (total pool). IL‐4 evaluated by enzyme‐linked immunosorbent assay was significantly higher in patients with CE compared to those without (NO‐CE subjects) when whole‐blood was stimulated with AgB1 and with the total pool. Moreover, IL‐4 levels in response to the total pool were significantly increased in patients with active cysts. Receiver Operator Curve analysis identified a cut‐off point of 0.59 pg/mL predicting active cysts diagnosis with 71% sensitivity and 82% specificity in serology‐positive CE patients. These data, if confirmed in a larger cohort, offer the opportunity to develop new diagnostic tools for CE based on a standardized source of AgB as the peptides.

## 1 INTRODUCTION

Cystic echinococcosis (CE) is a widespread chronic zoonosis caused by the parasite *Echinococcus granulosus* sensu *lato*. In humans, the parasite forms cysts mainly in the liver causing severe complications. An estimated 1.2 million people are infected worldwide.^1^ and Italy and the Mediterranean countries are considered endemic.^2^, ^3^ In Italy, the prevalence in 2014, estimated on hospital discharge records, is up to 1.06/100 000 inhabitants/year,^4^ but it is expected that these data are underestimated as outpatients or asymptomatic patients are not included in the analysis.^5^ CE diagnosis and clinical management are based on ultrasound (US). Serology complements imaging, but current tests present low sensitivity (up to 25% negative results).^6^ Moreover, serology does not allow assessing cyst biological viability, cross‐reactivity exists and may remain positive after cure, or negative after reactivation,^7^, ^8^ causing the need of long follow‐up to establish the biological viability loss of the cyst. Indeed, not always cyst stage, evaluated by imaging, coincides with cyst biological viability. This is the case of CE3a cysts, having equal probability of being viable or non‐viable, and of CE4 cysts, as a proportion of imaging‐inactive cysts are biologically viable when CE4 stage is the result of treatment.^9^, ^10^, ^11^ Therefore, improved diagnostic systems and identification of biomarkers for CE diagnosis and follow‐up are needed.

Among *E. granulosus* compounds, antigen B (AgB), one of the most abundant antigens of hydatid fluid, has been extensively studied.^12^, ^13^, ^14^, ^15^, ^16^, ^17^, ^18^ AgB is a multimeric protein of 8 KDa subunits encoded by a multigene family.^19^, ^20^, ^21^ To date, five subunit genes have been identified^22^; however, several aspects of AgB‐subunit composition and oligomeric structure are poorly characterized. Moreover, AgB subunits could be differentially expressed within individuals and/or throughout the parasite life cycle.^23^ Therefore, the composition of native‐AgB is uncertain and may have a high degree of variation influencing its use in diagnosis. In such scenario, it is essential to assess the immunogenicity and the diagnostics potentials of each AgB‐subunit.

So far, studies on the diagnostic value of recombinant AgB subunits have been focused on AgB1 or AgB2.^24^, ^25^ The performance of all AgB subunits for serological diagnosis of CE was recently evaluated with a reported reactivity order of AgB1 >  AgB4 >  AgB2 >  AgB5 >  AgB3.^26^ Several studies assessed the antigenicity of selected synthetic peptides derived from AgB1 and AgB2 subunits and among them, p65, p89‐122, p176, Gu4 were promising as diagnostic reagents.^27^, ^28^, ^29^ Synthetic peptides have been used as antigens for the diagnosis of parasitic diseases as malaria, leishmaniasis and schistosomiasis.^30^ Indeed, they show several advantages over native or recombinant antigens being well characterized, easily standardized and producible in large amounts, cost‐saving, highly pure and endotoxin‐free.

Alternative laboratory tests for the diagnosis of chronic infections as tuberculosis or toxoplasmosis are based on cytokines detection upon stimulation of whole blood with pathogen‐derived molecules, including antigenic peptides.^31^, ^32^, ^33^, ^34^ We recently set‐up a whole‐blood assay based on interleukin (IL)‐4 detection in response to native‐AgB which showed a good accuracy for CE diagnosis and staging.^12^

Thus, we set‐up a pilot study to: (i) analyse the IL‐4 response to multiepitope synthetic peptides spanning the immunodominant regions of the 5 AgB isoforms and (ii) determine if the selected peptides may increase the diagnostic accuracy of the whole‐blood assay for CE diagnosis and follow‐up.

## 2 MATERIALS AND METHODS

### 2.1 Study population

Patients admitted to the National Institute of Infectious Diseases (INMI) and Sant’ Andrea Hospital between March 2015 and December 2016 with suspected CE [risk factors for CE at the interview^13^ and the presence of cysts at the time of the visit] were evaluated for enrolment.

CE was diagnosed based on the images characteristics, and serology as confirmatory test. Therefore, both serology‐positive and serology‐negative pathognomonic imaging‐positive patients were included. At INMI, serological diagnosis of *E. granulosus* was performed using IgG enzyme‐linked immunosorbent assay (ELISA) (Cypress Diagnostic, Langdorp, Belgium) and Western blot IgG (Euroimmun Labordiagnostika, Luebeck, Germany); at the Sant’ Andrea Hospital by indirect hemagglutination (IHA Cellognost Echinococcosis; Dade Behring, Newark, NJ, USA) and a home‐made Western blot IgG.

Patients with cysts in the liver or any other location were included (Table 1). Hydatid cysts were staged according to WHO classification.^35^, ^36^ Patients were classified as having “active cysts” (CE1, CE2), “inactive cysts” (CE4, CE5) and “transitional‐cysts” (CE3a, CE3b). Patients having multiple cysts were classified according to the more active stage.^9^ Metacestodes viability was not performed in our Institutions as the common procedures to evaluate it are limited by practical and ethical reasons and proton magnetic resonance spectroscopy was not available. A previous correlation of the cyst stages (defined by US) with their biological viability was considered to classify patients with CE.^9^ Based on this evaluation, CE1, CE2 and CE3b are considered viable cysts, CE3a has equal probability of being viable or non‐viable, and inactive cysts (CE4 and CE5) are usually non‐viable, even if some CE4 cysts have been described as viable.^9^

**Table 1:** Demographical and clinical characteristics of the enrolled subjects

	Patients with CE	NO‐CE subjects
N (%)	43 (100.0)	26 (100.0)
Median Age years (IQR)	46 (34‐61)	53 (47‐69)
Female gender N (%)	19 (44.2)	13 (50.0)
Origin N (%)		
Italy	30 (69.8)	22 (84.6)
Eastern Europe	11 (25.6)	2 (7.8)
Africa	1 (2.3)	‐
Asia	‐	1 (3.8)
North America	‐	1 (3.8)
South America	1 (2.3)	‐
Serology‐positive results N (%)	26 (60.5)	0 (0)^a^
Previous Treatment N (%)	27 (62.8)	‐
Present Treatment N (%)	16 (37.2)	‐
Cyst localization N (%)		
Liver	27 (62.8)	14 (77.8)
Lung	3 (7.0)	1 (5.5)
Liver and Lung	5 (11.6)	‐
Other localization	8 (18.6)	3 (16.7)
Patients with active cysts N(%)	16 (37.2)	‐

a serology performed in 18 (69.2%) control subjects.

N, Number; IQR, Interquartile Range; y, year; US, Ultrasound.

“NO‐CE” subjects, included as controls, were healthy volunteers or subjects enrolled with suspected CE who had a CE diagnosis excluded by US, by clinical evaluation and serology.

Demographic and laboratory data, symptoms, treatment and cyst descriptions were collected.

Ethical Committees of INMI (parere 34/2010; 28/2014) and Sant'Andrea Hospital (Prot. C.E. n. 436/11) approved the study; enrolled individuals provided written informed consent.

### 2.2 AgB synthetic peptides selection

Sequences of the “*E. granulosus*” (G1) AgB subunits were obtained by search in Genebank database. Ninety‐nine full‐length and partial sequences were obtained as follows: 18 for AgB1, 32 for AgB2, 18 for AgB3, 20 for AgB4 and 11 for AgB5. Consensus sequence for each AgB‐subunit was generated by multiple alignment, as described.^37^, ^38^, ^39^

The consensus sequences of the AgB subunits shared a large degree of sequence homology. In all the AgB subunits, three main areas containing HLA‐II promiscuous multi‐epitopic areas were identified, and for each area, one or more peptides were designed. Epitope recognition and specificity to *E. granulosus* were assessed by BLAST analysis. Each selected peptide contains epitopes binding HLA‐DR alleles belonging to at least three different HLA‐DR supertypes.^40^ Overall, for each AgB‐subunit the HLA‐DR allelic variants allow a coverage of more than 90% of the most frequent HLA‐DR alleles present in the human populations.^41^

Designed peptides were synthesized by standard Fmoc chemistry (INBIOS, Naples, Italy) and purified by preparative HPLC at 90% purity. Peptides corresponding to the same AgB‐subunit were pooled. AgB peptide pools are listed in Table 2.

**Table 2:** List of AgB peptides

Peptide	Position	Sequence	AgB protein
Peptide AgB1_1	MRFCLLLALALVSFVVVTQADDGLT	(1‐25)	B1
Peptide AgB1_2	SVMKMFGEVKYFFERDPLGQKV	(30‐51)	B1
Peptide AgB1_3	EVFQLLRKKLRMALRSHLRGLIAEGE	(61‐85)	B1
Peptide AgB2_1	MRTYILLSLALVAFVTVVQAKDEP	(1‐24)	B2
Peptide AgB2_2	GELRDFFRNDPLGQRLVALGNDLTAICQK	(36‐64)	B2
Peptide AgB2_3	QKLQLKIREVLKKYVKNLVEEKDD	(63‐86)	B2
Peptide AgB3a_1	MKFCMLLALALVSFVVVARAECDDD	(1‐25)	B3
Peptide AgB3a_2	KDFFRRDPLGKKLVEVMKEVASV	(42‐64)	B3
Peptide AgB3a_3	CEMVRKKARMALKAYVRKLIEEAE	(65‐88)	B3
Peptide AgB3b_1	MKFCMLLALALVSFVVVARADDDD	(1‐24)	B3
Peptide AgB4_1	MRTYILLSLALVAFVAVVQAKAEP	(1‐24)	B4
Peptide AgB4_2	RDFFRSDPLGQKLVALGRDLTAICQK	(39‐64)	B4
Peptide AgB4_3	LQLKVHEVLKKYVKDLLEEEDE	(65‐86)	B4
Peptide AgB5_1^a^	LALVSFVAVARAECDD	(3‐17)	B5
Peptide AgB5_2^a^	KDFFRRDPLGKKLVEVMKEVASV	(35‐57)	B5
Peptide AgB5_3^a^	EMVRKKARMALKAYVRKLIEEAE	(59‐81)	B5
Peptide AgB5/1_2:	KEFFASDPMGQKL	(20‐32)	B5
Peptide AgB5/1_3:	KDFFLLARTKARSALRDYVKRLMDE	(40‐64)	B5

a peptide synthesis failed.

### 2.3 Stimuli

Peptide pools were used at 1 or 10 ug/mL peptide; native‐AgB at 1 ug/mL [produced by EP]; staphylococcal enterotoxin B (SEB) (Sigma, St Louis, MO, USA) at 200 ng/mL, was used as positive control.

### 2.4 Whole‐blood assay

Five‐hundred microlitre of heparinized whole‐blood per well was seeded in a 48‐well plate (Corning Costar, New York, NY, USA) with or without the above‐listed stimuli and incubated at 37°C (5%CO2) for 20‐24 hour. Plasma was harvested and stored at −80°C.

### 2.5 Cytokine determination

ELISA was used to detect IL‐4 (Quantikine HS IL‐4 ELISA, R&D Systems) following the manufacturer's instructions. Range of detection was as follows: 0.25‐16 pg/mL. IL‐4 values were subtracted from the unstimulated control.

### 2.6 Statistical analysis

Analysis was carried out with SPSS v.19 for Windows (SPSS Italia SRL, Bologna, Italy) and Prism 6 software (Graphpad Software 6.0, San Diego, MO, USA). Medians and interquartile ranges (IQR) were calculated for continuous measures; Chi‐square for dichotomous measures. The Mann–Whitney U test was used for pairwise comparisons. The cut‐off values were defined by a Receiver Operator Characteristic analysis (ROC). Test concordance was assessed by k‐statistics where k ≤ 0.20 was considered “slight”, 0.20 <  k ≤ 0.40 “fair”, 0.40 <  k ≤ 0.60 “moderate”, 0.60 <  k ≤ 0.80 “substantial” and 0.80 <  k ≤ 1.00 “optimal.”

## 3 ReSults

### 3.1 Study population

We enrolled 69 CE‐suspect subjects, 43 (62.3%) had a confirmed CE diagnosis, whereas 26 (37.7%) were classified, after the clinical investigation, as “NO‐CE subjects” (Table 1). Sixteen patients with CE (37.2%) had active cysts (CE1 and CE2), 4 (9.3%) transitional‐cysts [2 (50%) had CE3a cysts and 2 (50%) had CE3b cysts] and 23 (53.5%) inactive cysts (CE4 and CE5). Among the 23 patients with inactive cysts, eight had spontaneously inactivated cysts, whereas the remaining patients (15) reached inactivation after therapy. Therefore, we included only patients in whom the minimum time between the last treatment and enrolment was at least of 4 years (20 patients). NO‐CE group included 18 (69.2%) subjects with abdominal or lung cysts not related to CE and 8 (30.8%) healthy donors. Demographic and clinical features are detailed in Table 1.

### 3.2 IL‐4 response to AgB1 subunit pool and total peptide pool is significantly associated with CE

To evaluate the most appropriate experimental setting for detecting IL‐4‐specific response, stimulation with several concentrations of AgB peptide pools was assessed in 13 patients with CE. The highest IL‐4 production was measured in whole‐blood stimulated with the AgB1 pool, the AgB2 pool and with the total pool at 1ug/mL, or with the AgB4 pool at 10ug/mL (data not shown). The AgB3 and the AgB5 peptide pools did not induce IL‐4 production, and therefore, they were excluded from the subsequent analysis (data not shown).

**Figure 1:**
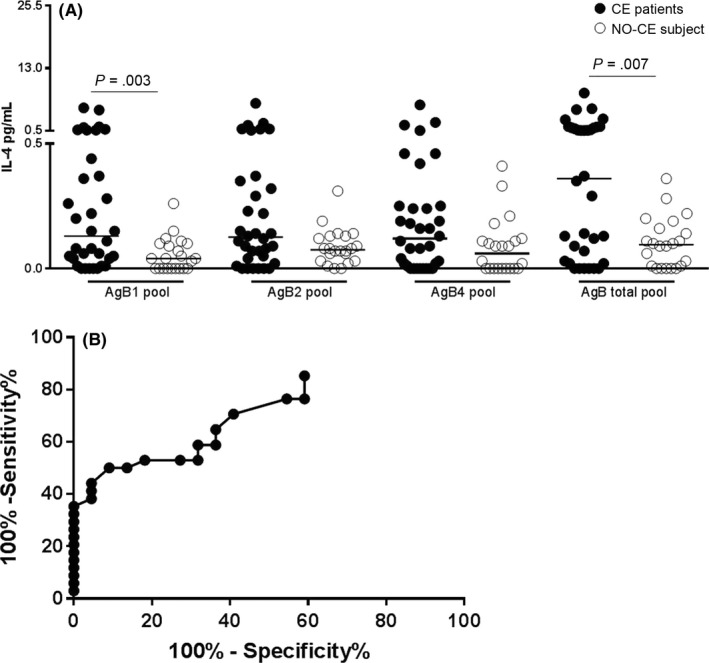
Increased whole‐blood IL‐4 response to AgB1 and total pools is significantly associated with CE. (A) IL‐4 levels are significantly increased in patients with CE (black dots) compared to NO‐CE subjects (white dots) (*P* = .003 and *P* = .007, respectively). (B) Significant area under curve (AUC) analysis results were obtained for AgB1 (AUC, 0.73; P = .004). The cut‐off point of 0.27 pg/mL predicted CE with 35% sensitivity and 100% specificity. Horizontal bars represent medians. IL‐4 concentrations were determined by ELISA. Responses were compared using the Mann–Whitney test; differences were considered significant at *P*‐values of ≤ .05

Due to the limited amount of blood recovered, the initial analysis was performed on 34 patients with CE and 22 NO‐CE subjects (Figure 1). AgB1 pool or AgB total pool induced high levels of IL‐4, and a significant difference was found between the patients with CE and the NO‐CE subjects (*P* = .003, *P* = .007 respectively; Figure 1A). Therefore, we performed a ROC analysis to evaluate the potential use for CE diagnosis of the whole‐blood assay based on AgB1 pool or AgB total pool. We found significant results in the area under curve for AgB1 (AUC) analysis (AUC, 0.73; 95% confidence interval (CI), 0.59‐0.86, *P* = .004) (Figure 1B). For scoring purposes, a cut‐off value was chosen to maximize the sum of sensitivity and specificity. The AgB1 pool cut‐off point of 0.27 pg/mL predicted CE with 35% sensitivity (95% CI, 19.8%‐53.5%) and 100% specificity (95% CI, 84.6%‐100%; Table 3a). A significantly higher proportion of positive results was found among patients with CE (11/34; 32.4%) compared to “NO‐CE” subjects (0/22; 0%) (*P* ≤ .0001).

**Table 3:** (a) Sensitivity and Specificity of serology and whole‐blood test for CE diagnosis and (b) active‐cysts diagnosis

Antigen	Whole‐blood cut‐off value (pg/mL)	Se (%) (positive over total)	Sp (%) (positive over total)
(a)			
Serology		65 (26/40)	100 (0/18)
Native‐AgB	0.28	55 (22/40)	96 (1/26)
AgB total pool	0.29	53 (21/40)	96 (1/26)
AgB1	0.27	35 (12/34)	100 (0/22)

Antigen	Subjects tested	Whole‐blood cut‐off value (pg/mL)	Se (%) (positive over total)	Sp (%) (positive over total)
(b)				
Serology	All	‐	78 (14/18)	45 (9/20)
Native‐AgB	All	0.42	61 (11/18)	75 (15/20)
AgB total pool	All	0.59	61 (11/18)	85 (17/20)
AgB total pool	“serology‐positive” subjects	0.59	71 (10/14)	82 (9/11)
AgB total pool	“serology‐negative” subjects	0.34	25 (1/4)	78 (7/9)

AgB: Antigen B; Se: Sensitivity; Sp: Specificity.

**Figure 2:**
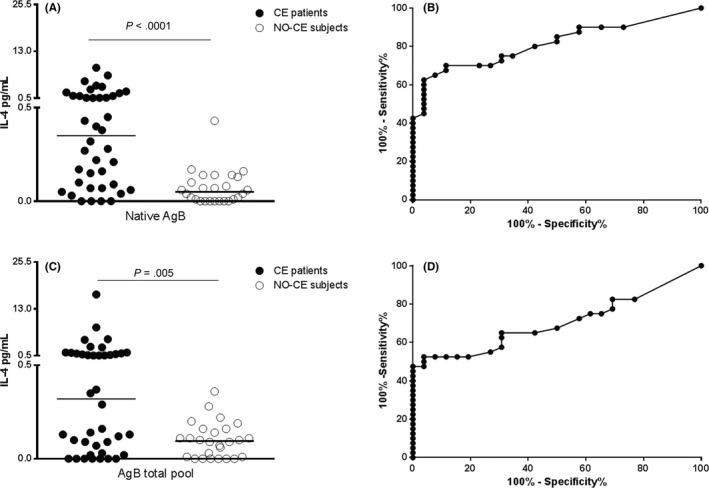
Comparison of the whole‐blood IL‐4 response to native‐AgB and AgB total pool between patients with CE and NO‐CE subjects. (A) IL‐4 levels in response to native‐AgB are significantly increased in patients with CE (black dots) compared to NO‐CE subjects (white dots) (*P* < .0001). (B) Significant area under the curve (AUC) analysis results were obtained for native‐AgB (AUC, 0.77; *P* = .0002). The cut‐off point of 0.28 pg/mL predicted CE with 55% sensitivity and 96% specificity. (C) IL‐4 levels in response to AgB total pool are significantly in patients with CE (black dots) compared to NO‐CE subjects (white dots) (*P* = .005). (D) Significant AUC analysis results were obtained for AgB total pool (AUC, 0.69; *P* = .009). The cut‐off point of 0.29 pg/mL predicted CE diagnosis with 53% sensitivity and 96% specificity. Horizontal bars represent medians. IL‐4 concentrations were evaluated by ELISA. Responses were compared using the Mann–Whitney test; differences were considered significant at *P*‐values of ≤ .05

We compared the accuracy for diagnosing CE of the whole‐blood test based on AgB total pool to that based on the native‐AgB. As shown in Figure 2, the IL‐4 levels in response to native‐AgB (Figure 2A), as demonstrated,^12^ and to the AgB total pool (Figure 2C) were significantly associated with CE (*P* < .0001 and *P* = .005, respectively) in all the subjects enrolled (40 patients with CE and 26 NO‐CE subjects). Significant AUC analysis results were found for both antigens (AUC, 0.81; 95% CI, 0.71%‐0.92%, *P* < .0001; AUC, 0.70; 95% CI, 0.58‐0.83, *P* = .006, respectively) (Figure 2B,D), and a cut‐off value was chosen. The native‐AgB cut‐off point of 0.28 pg/mL predicted CE with 55% sensitivity (95% CI, 38.5%‐96.2%) and 96% specificity (95% CI, 80.4%‐99.9%) and the AgB total pool cut‐off point of 0.29 pg/mL predicted CE with 53% sensitivity (95% CI, 36.1%‐68.5%) and 96% specificity (95% CI, 80.4%‐99.9%; Table 3a). A significantly higher proportion of positive results was found among patients with CE (21 of 40; 52.5%) compared to “NO‐CE” subjects (1/26; 3.8%) (*P* = .0002). The accuracy of the serology for the CE diagnosis was also evaluated, showing a 65% sensitivity and 100% specificity (Table 3a).

Overall, these data demonstrate that the AgB1 subunit is the more immunogenic AgB protein in our whole‐blood assay. Moreover, the IL‐4 response induced by the AgB total pool is similar to that induced by the native‐AgB.

### 3.3 IL4‐response to the AgB total pool is significantly associated with active cysts

We evaluated if the whole‐blood test based on the AgB peptide pools is useful for assessing cyst biological viability. We stratified the patients with CE according to their cyst viability: “active‐cysts” group, including CE1, CE2 and CE3b, and the “inactive‐cysts” group including CE4 and CE5. The CE3a cysts group was excluded from the analysis due to its small sample size. The initial analysis was performed on 17 patients with active cysts and 16 patients with inactive cysts.

**Figure 3:**
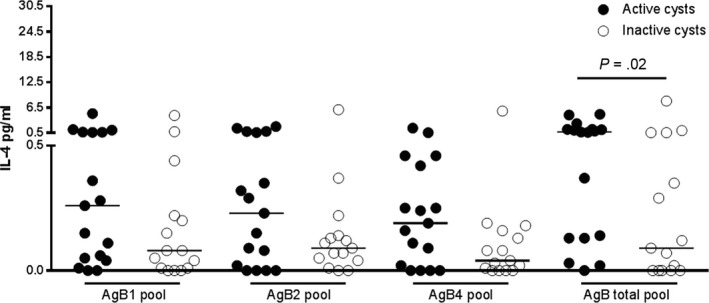
The IL‐4 levels in response to AgB total pool are increased in patients with active cysts. IL‐4 levels are increased in CE patients with active cysts (black dots) compared to patients with inactive cysts (white dots) (*P* = .02). Horizontal bars represent medians. IL‐4 concentrations were determined by ELISA. Responses were compared using the Mann–Whitney test; differences were considered significant at *P*‐values of ≤ .05

Increased IL‐4 levels were found between the “active” and the “inactive‐cysts” groups (*P* = .02) in response to AgB total pool (Figure 3).

**Figure 4:**
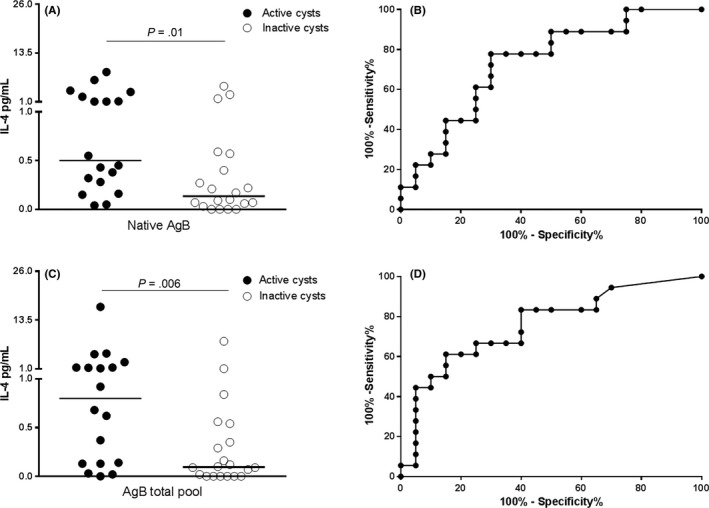
Comparison of the whole‐blood IL‐4 response to native‐AgB and AgB total pool between CE patients with active and inactive cysts. (A) IL‐4 levels in response to native‐AgB are significantly increased in CE patients with active cysts (black dots) compared to patients with inactive cysts (white dots) (*P* = .01). (B) Significant area under the curve (AUC) analysis results were obtained for native‐AgB (AUC, 0.77; *P* = .005). The cut‐off point of 0.42 pg/mL predicted CE with 61% sensitivity and 75% specificity. (C) IL‐4 levels in response to AgB total pool are significantly in CE patients with active cysts (black dots) compared to patients with inactive cysts (white dots) (*P* = .006). (D) Significant AUC analysis results were obtained for AgB total pool (AUC, 0.77; *P* = .004). The cut‐off point of 0.59 pg/mL predicted CE diagnosis with 61% sensitivity and 85% specificity. Horizontal bars represent medians. IL‐4 concentrations were determined by ELISA. Responses were compared using the Mann–Whitney test; differences were considered significant at *P*‐values of ≤ .05

Based on this result, we compared the accuracy for diagnosing active cysts of the whole‐blood test based on AgB total pool or native‐AgB in all the patients enrolled. IL‐4 levels in response to native‐AgB (Figure 4A) and to the AgB total pool (Figure 4C) were significantly associated with active cysts (*P* = .01 and *P* = .006, respectively). Therefore, we performed an additional ROC analysis between the “active” and “inactive‐cysts” groups. Significant AUC analysis results were found for both antigens (AUC, 0.73; 95% CI, 0.57‐0.89, *P* = .01; AUC, 0.76; 95% CI, 0.6‐0.91, *P* = .007, respectively) (Figure 4B,D), and a cut‐off value was chosen. The native‐AgB cut‐off point of 0.42 pg/mL predicted active cysts with 61% sensitivity (95% CI, 35.8%‐82.7%) and 75% specificity (95% CI, 50.9%91.3%) and the AgB total pool cut‐off point of 0.59 pg/mL predicted active cysts with 61% sensitivity (95% CI, 35.6%‐82.7%) and 85% specificity (95% CI, 62.1%‐96.8%) (Table 3b). A significantly higher proportion of positive results was found among patients with active cysts (11 of 18; 61.1%) compared to those with inactive cysts (three of 20; 15.0%) (*P* = .003).

These data suggest that, similarly to the native‐AgB, the IL‐4 response to the AgB total pool is associated with active cysts.

### 3.4 Agreement between experimental whole‐blood results and serology

We evaluated the agreement between the whole‐blood assay based on native‐AgB or AgB total pool and the commercial serology tests used for CE diagnosis.

The agreement between the whole‐blood test based on the native‐AgB and the serology in all the enrolled subjects was fair (k = 0.4; *P* = .002) (Table S1). These results depend on the high proportion of whole‐blood negative results (62.5%), mainly in patients with inactive cysts as previously found.^12^

Considering all the patients with CE and NO‐CE subjects, the agreement between the experimental test based on AgB total pool and the commercial serology was moderate (k = 0.5; *P* < .0001; Table 4). Similarly, considering the patients with CE according to cyst stage, in patients with active cysts, the agreement between the two tests was moderate (k = 0.5; *P* = .05, Table 4), whereas among patients with inactive cysts, it was fair and not significant (Table 4).

**Table 4:** Concordance between serology and whole‐blood assay based on total pool for the diagnosis of CE (cut‐off: 0.29)

	N	Serology positive/whole‐blood positiveN (%)	Serology positive/whole‐blood negativeN (%)	Serology negative/whole‐blood positiveN (%)	Serology negative/whole‐blood negativeN (%)	K	Concordance	*P* value^a^
NO‐CE subjects	18	0 (0)	0 (0)	1 (5.6)	17 (94.4)	‐	0.94	‐
Active cysts	18	11 (61.1)	3 (16.7)	1 (5.5)	3 (16.7)	0.5	0.78	.05
Inactive cysts	20	5 (25)	6 (30.0)	2 (10.0)	7 (35)	0.2	0.60	.28
All	56	16 (28.6)	9 (16.1)	4 (7.1)	27 (48.2)	0.5	0.77	**<.0001**

a Pearson‐ Chi‐square test.

N, Number of patients; k= concordance test; CE, Cystic Echinococcosis.

Therefore, these data suggest that the results of a positive serology test coupled with a negative whole‐blood test are likely indicative of inactive CE cysts stage. Indeed, the sensitivity/specificity of the whole‐blood test based on the AgB total pool was calculated, and in serology‐positive patients, a negative whole‐blood has a specificity of 71% for inactive‐cysts diagnosis; moreover, in serology‐negative patients, a positive whole‐blood result has a specificity of 78% for active‐cysts diagnosis (Table 5).

**Table 5:** Sensitivity and specificity of whole‐blood test based on AgB total pool for the diagnosis of active or inactive cysts (cut‐off: 0.59)

Total patients evaluated						Whole‐blood positive				Whole‐blood negative			
		Serology positive		Serology negative		Serology positive		Serology negative		Serology positive		Serology negative	
	N	Whole‐blood positiveN (%)	Whole‐blood negativeN (%)	Whole‐blood positiveN (%)	Whole‐blood negativeN (%)	Se (%)	Sp (%)	Se (%)	Sp (%)	Se (%)	Sp (%)	Se (%)	Sp (%)
NO‐CE subjects	18	0 (0)	0 (0)	0 (0)	18 (100)								
Active cysts	18	10 (55.5)	4 (22.2)	1 (5.6)	3 (16.7)	**71 (10/14)**	**82 (9/11)**	**25 (1/4)**	**78 (7/9)**	29 (4/14)	18 (2/11)	75 (3/4)	22 (2/9)
Inactive cysts	20	2 (10.0)	9 (45.0)	2 (10.0)	7 (35.0)	18 (2/11)	29 (4/14)	22 (2/9)	75 (3/4)	**82 (9/11)**	**71 (10/14)**	78 (7/9)	25 (1/4)
All	56	12 (21.4)	13 (23.2)	2 (3.6)	29 (51.8)								

Pearson‐ Chi‐square test.

N, Number of patients; k= concordance test; Se, Sensitivity; Sp, Specificity; CE, Cystic Echinococcosis.

### 3.5 IL4‐response to the AgB total pool identifies active cysts among CE patients with a positive serology

In our study, 26 of 43 (60.5%) patients with CE were serology positive; among them, 14 had active cysts, one had CE3a cysts, and 11 had inactive cysts. As mentioned before, CE3a samples or samples with follow‐up <4 years were excluded from the analysis. Therefore, the analysis was carried out on 25 patients with CE.

**Figure 5:**
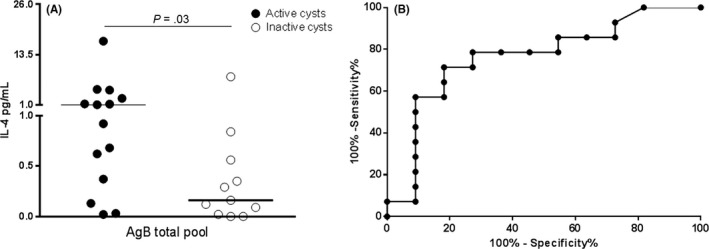
Whole‐blood IL‐4 response AgB total pool in CE patients with a positive serology associates with active cysts. (A) IL‐4 levels in response AgB total pool are significantly increased in CE patients with active cysts (black dots) compared to patients with inactive cysts (white dots) (*P* = .03). (B) Significant area under the curve (AUC) analysis results were obtained (AUC, 0.82; *P* = .006). The cut‐off point of 0.59 pg/mL predicted CE with 71% sensitivity and 82% specificity. Horizontal bars represent medians. IL‐4 concentrations were determined by ELISA. Responses were compared using the Mann–Whitney test; differences were considered significant at *P*‐values of ≤ .05

IL‐4 response to the AgB total pool was significantly higher in patients with active cysts compared to patients with inactive cysts (*P* = .03, Figure 5A). The ROC analysis between the “active” and “inactive cysts” groups showed significant AUC results (AUC, 0.76; 95% CI, 0.56‐0.96, *P* = .03; Figure 5B). The cut‐off point of 0.59 pg/mL predicted active cysts with 71% sensitivity (95% CI, 41.9%‐91.6%) and 82% specificity (95% CI, 48.2%‐97.7%; Table 2). According to this cut‐off, we found a significantly higher proportion of positive results among patients with active cysts (10 of 14; 71.4%) compared to those with inactive cysts (two of 11; 18.2%; *P* = .008). The accuracy of the serology for active‐cysts diagnosis was also evaluated, showing a 78% sensitivity and 45% specificity (Table 3a). Therefore, the whole‐blood test based on the AgB total pool may be useful, after a positive score to the serology, to identify the patients with active and biologically viable cysts.

## 4 DISCUSSION

CE diagnosis is based primarily on imaging examination and serological tests which, unfortunately, lack sensitivity^17^, ^18^ and do not provide information regarding the cyst biological viability. Recently, we showed that a T‐cell‐based test based on the detection of the IL‐4‐response to native‐AgB may have good potential for CE diagnosis and staging.

Native or recombinant antigens are powerful reagents for serology, because they contain a large spectrum of epitopes, which whilst guarantee their recognition theoretically by all individuals, may cause cross‐reactions, compromising the specificity of the system. For this reason, synthetic peptides are becoming standard reagents for T‐cell‐based assays as no conformation constrains are required for their recognition. Here, we analysed the immunogenicity of a panel of selected synthetic peptides spanning the immunogenic portions of 5 AgB subunits, and we showed that in whole‐blood assay (i) the AgB1 subunit is the more immunogenic AgB protein and (ii) they may be useful in a two‐step approach for the evaluation of cyst viability, after testing in longitudinal studies. Indeed, the IL‐4 response after specific stimulation with a pool of these peptides is significantly associated with CE and with the presence of active cysts with 71% sensitivity and 82% specificity in CE patients with a positive serology.

AgB is a 8KDa multimeric protein, and five subunits have been identified. So far, the immunogenicity and immunodiagnostic accuracies of AgB1 and AgB2 have been evaluated demonstrating that the AgB2 subunit provided the highest diagnostic sensitivity and specificity.^24^, ^25^ However, other studies reported a higher immunogenicity for the AgB1 subunit.^26^, ^42^ In line with these results, we found that AgB1 pool induced high levels of IL‐4 and a significant difference was found between the patients with CE and the NO‐CE subjects demonstrating that AgB1 is the most immunogenic AgB protein in our whole‐blood assay. Moreover, as previously shown^26^ the AgB3 or the AgB5 pools were poorly immunogenic. The low antigenicity of AgB3 may be related to its biochemical properties,^23^ which makes difficult the processing by the antigen‐presenting cells, or the inferior expression compared to the other AgB subunits.^43^

Besides the AgB1 pool, the AgB total pool induced high IL‐4‐levels, which are associated with CE, similarly to that induced by the native‐AgB. Moreover, this response was associated with active cysts, and its diagnostic accuracy for diagnosing active cysts is improved in CE patients with a positive serology. The comparison between the whole‐blood assay and the serology showed that, although the two tests have a similar accuracy for CE diagnosis, the whole‐blood test has a higher accuracy for identifying active cysts. Stratifying patients with CE according to cyst stage, we observed that the higher proportion of negative results to whole‐blood assay depended on the “inactive‐cysts” group, whereas about 67% of patients having active cysts scored positive to whole‐blood test.

These results imply several concepts. First of all, if CE4 and CE5 are biologically inactive, a weak immune response occurs. Indeed, although memory T cells would be surely generated when the cyst was active, terminally differentiated cells and exhaustion mechanisms may appear later leading to low immune responses. Moreover, as it is not clear the native‐AgB structure, the immune cells may be unable to respond to AgB because the in vitro stimulating antigen differs from the antigen that in vivo induced the immune response. Finally, in some cysts at early stage, their structure may prevent antigens release resulting in low/absent immune stimulation. Thus, as in both CE4‐CE5 and CE1 cases, the immune‐specific response is low, there is a low likelihood that the serology or the whole‐blood test score positive. On the other hands, as previously shown also for the native‐AgB,^12^ a positive serology score coupled to a negative score to whole‐blood is associated with inactive cysts.

Thus, although the whole‐blood assay based on AgB synthetic peptides showed a suboptimal diagnostic accuracy for CE diagnosis, it may provide information on the cyst biological viability, mainly in CE patients with a positive serology. We propose to use it after performing serology for CE diagnosis/monitoring, in a combined diagnostic test algorithm. Moreover, if these results are confirmed in larger cohorts, performing the whole‐blood test in serology‐positive patients could help in the clinical decision in those cases in which the cyst images have a low level of accuracy to define the cyst biological viability. Importantly, due to its association with the disease activity, the whole‐blood test may potentially identify cysts that are evolving through an active stage helping clinicians in cyst follow‐up.

Limitations of this study should be mentioned. First of all, a relatively small number of subjects was analysed. However, there are few reported cases of CE in our region.^2^ Therefore, the prospective enrolment of 43 patients with CE, together with 18 NO‐CE subjects enrolled as suspicious of CE, that were clinically characterized and studied by both experimental and routine approaches give strength to this pilot study. An additional limitation is the lack of testing of potential cross‐reactions with Alveolar Echinococcosis or Taeniasis/Cysticercosis. However, Alveolar Echinococcosis is absent in Italy, and Taeniasis/Cysticercosisis are diagnosed only in persons from endemic regions.^44^, ^45^ Moreover, different types of serological tests were used, as they are routine tests of the hospitals. Finally, the low number of patients with transitional‐cysts hampered us to draw any conclusion.

In conclusion, although the diagnostic accuracy of the whole‐blood test based on AgB synthetic peptides for diagnosing CE is suboptimal, this proof of concept contribute to generate improved and alternative tools using a standardized source of antigen for CE diagnosis and monitoring. Moreover, we showed the basis for the combination of a diagnostic algorithm based on serology followed by the whole‐blood test based on AgB synthetic peptides that is associated with active‐cysts identification. Further studies will validate this finding.

## CONFLICT OF INTEREST

The authors declare no conflict of interests.

## OBITUARY FOR MASSIMO AMICOSANTE

A great sense of loss and sorrow marked the recent death of Massimo Amicosante, who passed away in Sophia, Bulgaria on 1 October 2017 after an electric accident while he was playing guitar. He was only 49 yo. Massimo was a highly respected scientist of the respiratory and infectious diseases community, who devoted his life to understanding and designing experimental diagnostic tests for HIV, tuberculosis, berylliosis and Cystic Echinococcosis. He has been working in several countries mainly in Italy, United Kingdom, Bulgaria providing his expertise with intelligence, generosity, respect. Massimo will be remembered for a long period as a wonderful human being and with a lot of gratitude for his scientific expertise.
