# Supplementary material for: A T‐cell diagnostic test for cystic echinococcosis based on Antigen B peptides
Source: Parasite Immunol. 2017 Nov 24;39(12):e12499. doi: 10.1111/pim.12499 (PMC5846893; doi:10.1111/pim.12499)
Supplement: Supplementary file 1 [file PIM-39-na-s001.doc]

|  | **N** | **Serology positive/ whole-blood positive**  N (%) | **Serology positive/ whole-blood negative**  N (%) | **Serology negative/ whole-blood positive**  N (%) | **Serology negative/ whole-blood negative**  N (%) | **K** | **Concordance** | **P value^#^** |
| --- | --- | --- | --- | --- | --- | --- | --- | --- |
| NO-CE subjects | 18 | 0 (0) | 0 (0) | 1 (5.6) | 17 (94.4) | - | 0.94 | - |
| Active cysts | 18 | 12 (66.7) | 2 (11.1) | 2 (11.1) | 2 (11.1) | 0.4 | 0.78 | 0.13 |
| Inactive cysts | 20 | 3 (15.0) | 8 (40.0) | 3 (15.0) | 6 (30.0) | <0.01 | 0.45 | 0.77 |
| All**^¶^** | 56 | 15 (26.8) | 10 (17.9) | 6 (10.7) | 25 (44.6) | 0.4 | 0.71 | 0.002 |

**Supplementary Table 1. Concordance between serology and whole-blood assay based on native AgB for the diagnosis of CE**

**Footnote**: N: Number of patients; k= concordance test. #:Pearson- Chi-square test.
